# Rewiring of Gene Expression in *Pseudomonas aeruginosa* During Diauxic Growth Reveals an Indirect Regulation of the MexGHI-OpmD Efflux Pump by Hfq

**DOI:** 10.3389/fmicb.2022.919539

**Published:** 2022-06-23

**Authors:** Marlena Rozner, Ella Nukarinen, Michael T. Wolfinger, Fabian Amman, Wolfram Weckwerth, Udo Bläsi, Elisabeth Sonnleitner

**Affiliations:** ^1^Department of Microbiology, Immunobiology and Genetics, Max Perutz Labs, Vienna Biocenter (VBC), University of Vienna, Vienna, Austria; ^2^Molecular Systems Biology, Department of Functional and Evolutionary Ecology, Faculty of Life Sciences, University of Vienna, Vienna, Austria; ^3^Research Group Bioinformatics and Computational Biology, Faculty of Computer Science, University of Vienna, Vienna, Austria; ^4^Department of Theoretical Chemistry, University of Vienna, Vienna, Austria; ^5^Vienna Metabolomics Center, University of Vienna, Vienna, Austria

**Keywords:** *Pseudomonas aeruginosa*, Hfq, CRC, carbon catabolite repression, multiomics, norfloxacin, phenazines, MexGHI-OpmD efflux pump

## Abstract

In *Pseudomonas aeruginosa*, the RNA chaperone Hfq and the catabolite repression protein Crc act in concert to regulate numerous genes during carbon catabolite repression (CCR). After alleviation of CCR, the RNA CrcZ sequesters Hfq/Crc, which leads to a rewiring of gene expression to ensure the consumption of less preferred carbon and nitrogen sources. Here, we performed a multiomics approach by assessing the transcriptome, translatome, and proteome in parallel in *P. aeruginosa* strain O1 during and after relief of CCR. As Hfq function is impeded by the RNA CrcZ upon relief of CCR, and Hfq is known to impact antibiotic susceptibility in *P. aeruginosa*, emphasis was laid on links between CCR and antibiotic susceptibility. To this end, we show that the *mexGHI-opmD* operon encoding an efflux pump for the antibiotic norfloxacin and the virulence factor 5-Methyl-phenazine is upregulated after alleviation of CCR, resulting in a decreased susceptibility to the antibiotic norfloxacin. A model for indirect regulation of the *mexGHI-opmD* operon by Hfq is presented.

## Introduction

*Pseudomonas aeruginosa* (*Pae*) is associated with a broad spectrum of acute and chronic infections (Kerr and Snelling, 2009; Williams et al., 2010), and notorious for its resistance toward a variety of antibiotics (Pang et al., 2019). The pathogenic potential of *Pae* is based on its metabolic versatility, permitting fast adaptation to changing environmental conditions and to different niches such as soil, marine habitats, or different organisms (Gellatly and Hancock, 2013).

In *Pae*, the global regulator Hfq is involved in small RNA-mediated riboregulation as well as in carbon catabolite repression (CCR; Sonnleitner and Bläsi, 2014; Ferrara et al., 2015; Moreno et al., 2015; Sonnleitner et al., 2017). The function of Hfq in riboregulation of Gram-negative bacteria has been well studied. Hfq is known to stabilize sRNAs and to facilitate their annealing with target mRNAs (Santiago-Frangos and Woodson, 2018). Moreover, in *Pae*, Hfq was shown to bind directly to mRNAs that contain (AAN)_n_ or (ARN)_n_ repeats, in which A is an adenine, R is a purine (A/G), and N is any nucleotide. When present in the translation initiation region, these motifs can engage the tripartite binding pockets on the distal face of Hfq (Link et al., 2009; Malecka et al., 2021) to block translation during CCR (Sonnleitner and Bläsi, 2014). Hereby, the catabolite repression protein Crc acts as a co-repressor by stabilizing the Hfq-RNA complex (Sonnleitner et al., 2018; Pei et al., 2019; Malecka et al., 2021). Transcriptome analyses with PAO1*Δhfq* and PAO1*Δcrc* revealed many overlapping mRNA targets, regulation of which is governed by both, Hfq and Crc, during CCR (Sonnleitner et al., 2018). In this way, Hfq/Crc ensure a hierarchical use of nutrients by precluding the synthesis of uptake- and degradative functions required for metabolization of C/N sources other than the available preferred one(s), such as C4-dicarboxylates. Furthermore, a ChIP-seq approach showed that Hfq and Crc co-associate with nascent transcripts, whereas Crc was not observed on RNAs in the absence of Hfq (Kambara et al., 2018). These *in vivo* findings are in accord with previous results, showing that Crc is devoid of RNA binding activity (Milojevic et al., 2013), and that the association of Crc with RNA requires Hfq (Sonnleitner et al., 2018). Moreover, a RNA-seq based comparative transcriptome analysis of the *Pae* strains PAO1Δ*hfq* and PAO1Δ*hfq*Δ*crc* strongly suggested that Crc does not exert an independent regulatory function (Malecka et al., 2021).

Recent structural studies have revealed that Hfq/Crc complexes formed on individual mRNAs vary in form and composition (Dendooven, 2020). It is therefore conceivable that Hfq/Crc mediated regulation results in differential translational repression of target genes. This hypothesis may explain variations in translational control of individual genes in *Pae crc* deletion strains (Corona et al., 2018), which can result in a change of the order of metabolite utilization (La Rosa et al., 2016). In fact, the absence of Crc caused an unbalanced metabolism with poorly optimized metabolic fluxes in *P. putida* (La Rosa et al., 2015; Molina et al., 2019).

The response to different C-sources is coordinated by the RNA CrcZ (Sonnleitner et al., 2009; Valentini et al., 2014). CrcZ sequesters Hfq and/or Hfq/Crc when CCR is alleviated (Sonnleitner and Bläsi, 2014; Sonnleitner et al., 2018), which in turn leads to the metabolization of non- or less preferred C sources, such as mannitol. CrcZ expression is under control of the alternative sigma factor RpoN and the two-component system CbrA/B (Sonnleitner et al., 2009; Abdou et al., 2011). The signal responsible for CbrA activation remains unknown, but it is thought to be related to the internal energy status of the cell (Valentini et al., 2014). Furthermore, as CrcZ acts as a decoy for Hfq, CrcZ does not only interfere with direct translational repression by Hfq/Crc (Sonnleitner and Bläsi, 2014) but also with Hfq-mediated riboregulation by sRNAs (Sonnleitner et al., 2017). Moreover, Hfq-mediated regulation is linked to complex behavior including the control of virulence genes (Sonnleitner et al., 2003, 2006), quorum sensing (Kimyon et al., 2016; Yang and Lan, 2016), biofilm formation (Pusic et al., 2016) as well as antibiotic susceptibility (Pusic et al., 2018; Sonnleitner et al., 2020).

Most studies on Hfq/Crc mediated regulation in *Pae* have been performed during growth on specific carbon sources (Corona et al., 2018; Kambara et al., 2018; Sonnleitner et al., 2018; Molina et al., 2019). Comparatively, little is known on the dynamic rewiring of gene expression during diauxic growth in the simultaneous presence of a preferred carbon source, such as succinate, and a non-preferred one, such as mannitol (Valentini et al., 2014). Here, we aimed at obtaining a picture on the re-programing of regulatory networks and genes by using a multiomics approach, revealing changes in the transcriptome (RNA-seq), translatome (Ribo-seq), and proteome (MS, mass spectrometry). On the one hand, it was expected that these studies verify and validate transcripts subject to Hfq/Crc regulation during CCR. On the other hand, we anticipated that these studies reveal novel target genes that are differentially regulated after relief of CCR, i.e., after titration of Hfq/Crc by the RNA CrcZ, the levels of which are known to rise when the preferred carbon source is exhausted (Valentini et al., 2014). Here, we report an upregulation of the *mexGHI-opmD* operon encoding an efflux pump that expels the antibiotic norfloxacin and the virulence factor 5-Methyl-phenazine (5-Me-PCA). A model is presented for an indirect upregulation of the *mexGHI-opmD* operon after CrcZ mediated titration of Hfq.

## Materials and Methods

### RNA-seq and Ribo-seq Library Preparation and Analyses

**Figure 1:**
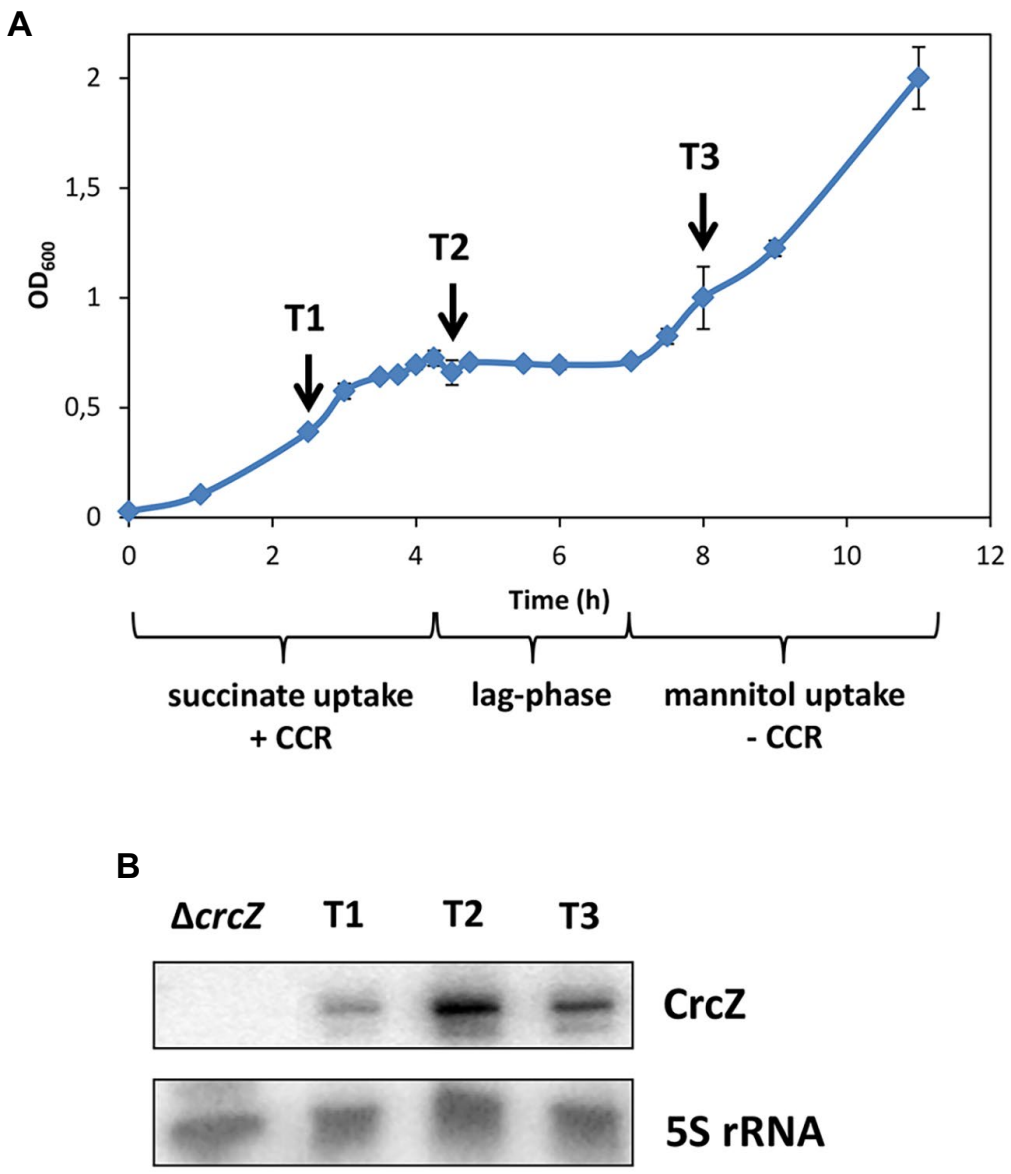
**(A)** Growth of strain PAO1 in Basal-Salt medium (BSM) supplemented with 5 mM succinate and 40 mM mannitol. The arrows indicate the times of sample withdrawal during carbon catabolite repression (CCR; T1), at the beginning of the lag-phase (T2) and after relief of CCR (T3). **(B)** The steady state levels of the regulatory RNA CrcZ were monitored by Northern blot analysis at T1, T2, and T3, respectively. 5S RNA was used as a loading control.

Three biological replicates of *P. aeruginosa* strain O1 (PAO1; Holloway et al., 1979) were grown at 37°C in 100 ml Basal-Salt medium [BSM; 30.8 mM K_2_HPO_4_, 19.3 mM KH_2_PO_4_, 15 mM (NH_4_)SO_4_, 1 mM MgCl_2_, and 2 μM FeSO_4_] supplemented with 5 mM succinate and 40 mM mannitol (Sonnleitner et al., 2009). Total RNA was prepared from each of the three biological replicates after they reached an OD_600_ of 0.4 [time 1 (T1)], 0.7 [time 2 (T2)], and 1.2 [time 3 (T3); Figure 1A], respectively. 10 ml samples were withdrawn from each culture, the cells were harvested by centrifugation at 4,000 *g* for 15 min at 4°C and frozen in liquid nitrogen. Total RNA was extracted using the TRIzol reagent (Ambion) according to the manufacturer’s instructions. DNA was removed using DNAse I (Roche), followed by phenol-chloroform purification and ethanol precipitation with 1/10 volume of 3 M NaOAc, pH 5.5. To deplete ribosomal RNAs, the Ribo-Zero™ Magnetic Kit for Gram-negative Bacteria (Epicentre) was used. The libraries were constructed using the NEBNext® Ultra™ Directional RNA Library Preparation Kit from Illumina. Around 100 base pair single end sequence reads were generated using the Illumina HiSeq V4 platform at the Vienna Biocenter Campus Science Support Facility.^1^ Quality control assessment, sequencing adapter removal, and mapping of the samples against the PAO1 reference genome (NCBI accession number NC_002516.2) were performed as described previously with Segemehl using default parameters (Hoffmann et al., 2009). Reads mapping to regions annotated as either rRNA or tRNA were discarded. The mapped sequencing data were prepared for visualization using the ViennaNGS tool box, and visualized with the UCSC Genome Browser (Wolfinger et al., 2015). Reads per gene were counted using BEDTools (Quinlan and Hall, 2010) and the Refseq annotation of *P. aeruginosa* (NC_002516.2). Differential gene expression analysis was performed with DESeq (Anders and Huber, 2010). The levels of transcripts with a fold-change (FC) greater than 2 and a multiple testing adjusted *value of p* below 0.05 were considered to be significant (shown in bold in Supplementary Table S1).

Ribosome profiling of elongating ribosomes (Ribo-seq; Ingolia et al., 2009) was performed with the same 100 ml cultures as used for the RNA-seq analyses. 10 ml samples were withdrawn from each culture after growth to an OD_600_ of 0.4 [time 1 (T1)], 0.7 [time 2 (T2)], and 1.2 [time 3 (T3); Figure 1A], respectively, transferred to new flasks and treated for 10 min with chloramphenicol (300 μg/ml) to stop translation. The cells were then harvested by centrifugation at 4,000 *g* for 15 min at 4°C, the pellet was washed in 50 ml ice cold lysis buffer (10 mM MgOAc, 60 mM NH_4_Cl, 10 mM TRIS–HCl, and pH 7.6), again pelleted by centrifugation at 5,000 *g* for 15 min at 4°C, and then frozen in liquid nitrogen. The pellets were re-suspended in 1 ml ice cold lysis buffer containing 0.2% Triton X-100, 100 μg/ml chloramphenicol, and 100 U/ml DNAse I, frozen in liquid nitrogen and cryogenically pulverized by repeated cycles of grinding in a pre-chilled mortar and freezing in a dry ice/ethanol bath. The lysates were centrifuged at 15,000 *g* for 30 min at 4°C to remove cellular debris. About 100 μl aliquots of the cleared lysates were treated with 4 μl of Micrococcal Nuclease (MNase, NEB) and 6 μl of the RiboLock RNase inhibitor (Thermo Scientific) for 1 h at 25°C with continuous shaking at 450 rpm. The lysates were layered onto 10%–40% linear sucrose density gradients in lysis buffer and centrifuged at 256,000 *g* for 3 h at 4°C. About 500 μl gradient fractions were collected by continuously monitoring the absorbance at 260 nm. The RNA was extracted from fractions containing 70S ribosomes with phenol-chloroform-isoamyl alcohol (25:24:1), and precipitated with ethanol. The samples were treated with DNase I (TURBO™ DNase, Thermo Scientific) and separated on a 15% polyacrylamide gel containing 8 M urea. Ribosome protected mRNA fragments ranging in size from 20 to 40 nucleotides were removed and eluted from the polyacrylamide gel by overnight incubation in elution buffer (0.3 M NaOAc, 1 mM EDTA) at 4°C, which was followed by another phenol-chloroform-isoamyl alcohol (25:24:1) extraction and ethanol precipitation. The quality of the RNA samples was subsequently analyzed with a 2100 Bioanalyzer and an Agilent RNA 6000 Pico Kit (Agilent Technologies). The RNA was further processed into cDNA libraries with NEBNext™ Small RNA Library Prep Set for Illumina® and their quality was assessed with the 2100 Bioanalyzer and a High Sensitivity DNA Kit (Agilent Technologies). Pipin Prep™ was used to purify the 140–160 bp cDNA products which corresponded to adapter-ligated 20–40 nucleotide long ribosomal footprints. RNA sequencing and data processing were performed as described above. The raw RNA-seq and Ribo-seq data were deposited in the ENA under accession number PRJEB52258.

### Extraction of Cellular Proteins

Cellular proteins were extracted from the same 100 ml cultures of two biological replicates as used for the RNA-seq and Ribo-seq analyses. 10 ml samples were withdrawn from each culture after growth to an OD_600_ of 0.4 [time 1 (T1)], 0.7 [time 2 (T2)], and 1.2 [time 3 (T3); Figure 1A], respectively, transferred to new flasks and treated for 10 min with chloramphenicol (300 μg/ml) to stop translation. The cells were harvested by centrifugation at 4,000 *g* at 4°C for 15 min and frozen in liquid nitrogen. The pellets were re-suspended in 200 μl 1x Laemmli buffer per 0.5 OD_600_ unit. The cell suspensions were sonicated for 1 min at 40% power for 40 cycles (Bandelin Sonopuls GM70), and then boiled for 5 min at 95°C. The cell debris was removed by centrifugation at 24,000 *g* for 10 min at room temperature. The samples (100 μg/lane) were loaded onto a 12.5% SDS polyacrylamide gel, which was stained with colloidal coomassie stain (Roth). The gel slices were cut into pieces and placed in 1.5 ml microcentrifuge tubes. About 1 ml of 200 mM ammonium bicarbonate (AmBic) in 50% acetonitrile (ACN) was added to each sample followed by incubation for 1 h at 37°C. The supernatant was discarded and the samples were further incubated with 500 μl of 50 mM AmBic in 5% ACN for 15 min at 37°C. The solution was removed and the gels slices were incubated two times in 500 μl of 100% ACN for 10 min at 37°C to remove water. The dried proteins were digested with 100 μl of trypsin (Thermo; 25 ng/μl), re-suspended in 25 mM AmBic in 10% ACN and 5 mM CaCl_2,_ and further incubated for 16 h at 37°C. The peptides were extracted with 150 μl of 50% ACN/1% of formic acid (FA) by sonication in a low intensity ultrasound bath for 3 min, after which the supernatants were transferred to 1.5 ml Eppendorf tubes. The gel was further incubated with 100 μl 90% ACN/1% FA for 15 min at room temperature. The supernatant was transferred to the same tubes as in the previous step and dried in a vacuum concentrator. The protein digests were desalted with C18 solid phase extraction (SPE; Agilent Technologies) and carbon graphite SPE as described by Furuhashi et al. (2014). After desalting, the corresponding eluates were pooled and dried in a vacuum concentrator.

### Liquid Chromatography-Mass Spectrometry Analysis

The samples were analysed with an Orbitrap Elite hybrid ion trap-orbitrap mass spectrometer (MS; Thermo Fisher Scientific) coupled to an UltiMate 3000 RSLCnano system (Thermo Fisher Scientific). The peptides were re-suspended in 99% of ACN and 1% of FA to a final concentration of 0.2 μg/μl and separated on a 50 cm EASY-Spray C18 LC column (Thermo Fisher Scientific). The peptides were eluted with a 120 ml gradient from 2 to 40% of solvent B (90% ACN, 0.1% FA) at a flow rate of 300 μl/min. Full scans were obtained with an Orbitrap mass analyzer. The scan range was set to 350–1800 Th, the resolution to 120,000 at 400 m/z, the AGC target to 1,000,000 ions, and the maximum injection time to 100 ms. Up to 20 most intense precursor ions (charge ≥ 2) were selected for CID fragmentation (normalized collision energy 35%, activation time 10 ms, isolation width 2.0 Th, and minimum signal threshold 1,000) in an ion trap. The MS2 AGC target was set to 5,000 ions and the maximum injection time to 50 ms. The dynamic exclusion of fragmented precursors was set to 30 s.

### Peptide Identification and Quantification

The peptide identification and protein quantification was done with MaxQuant (version 1.5; Cox and Mann, 2008; Cox et al., 2011). The spectra were searched against the *P. aeruginosa* protein database.^2^ The following settings were used: maximum two missed cleavages were allowed, methionine oxidation and protein N-terminal acetylation were set to variable modifications and mass tolerance for precursor mass was set to 4.5 ppm and for fragment masses to 0.6 Da. The maximum FDR for peptides and proteins was 1%. Match between runs function was used with 20 and 0.7 min alignment and matching time window, respectively. Protein quantification was done with a peptide count ≥2. Filtering and further data processing and statistics were done with Perseus 1.5 software and Excel.

### Northern Blot Analyses

Total RNA was isolated from strain PAO1 at OD_600_ = 0.4 (T1), 0.7 (T2), and 1.2 (T3) during diauxic growth in BSM medium supplemented with succinate (5 mM) and mannitol (40 mM), from strain PAO1 (pME10011) after growth to an OD_600_ of 2.0 in BSM medium supplemented with succinate (40 mM) and mannitol (40 mM), respectively, as well as from strains PAO1 (pME10011; pMMB67HE) and PAO1 (pME10011; pMMB*crcZ*) after growth to an OD_600_ of 2.0 in BSM medium supplemented with succinate (40 mM). The corresponding RNAs were purified using the TRIZol reagent (Ambion) according to the manufacturer’s instructions. The levels of the CrcZ RNA and 5S RNA (control) were determined by Northern-blotting using 5 μg of total RNA. The RNA samples were resuspended in 1x RNA loading dye (95% formamide, 10 mM EDTA pH 8.0, 0.025% xylene cyanol FF, and 0.025% bromophenol blue), denatured for 5 min at 98°C, separated on 6% polyacrylamide/8 M Urea gel, and then transferred onto a nylon membrane (Amersham) by electro-blotting. The RNAs were cross-linked to the membrane by exposure to UV light. The membranes were hybridized with gene-specific ^32^P-end-labeled oligonucleotides [CrcZ: K3 (5’GCT GGA GTC GTT ACG TGT TG-3`); 5S rRNA: I26 (5`-CCC CAC ACT ACC ATC GGC GAT GCG TCG-3`); Sonnleitner et al., 2018]. The hybridization signals were visualized using a PhosphoImager (Molecular Dynamics). The normalization was performed with ImageQuant TL version 8.1.

### Determination of the Minimal Inhibitory Concentration

PAO1 was grown to an OD_600_ of 0.3–0.4 in BSM supplemented with 40 mM succinate and mannitol, respectively. 2 ml of the cultures were transferred to 10 ml glass tubes, containing 50 μl norfloxacin in serial dilutions (concentration range 78 μg/L–10 mg/L). The cultures were shaken for 16 h at 37°C followed by measurement of the OD_600_. The MIC corresponds to the lowest concentration of antibiotics that impacted growth.

### *Candida albicans* Bioassay

300 μl of overnight culture of *C. albicans* was mixed with 10 ml YPD agar medium [2% Yeast Extract (Sigma-Aldrich), 4% peptone (Sigma-Aldrich), 4% Dextrose (Sigma-Aldrich), and 0.75% Agar (Merk)], poured into Petri dishes and incubated for 36 h at 37°C. 10 μl of the PAO1 culture grown in BSM (see above) supplemented with 40 mM succinate or 40 mM mannitol (OD_600_ = 3.0) were spotted on top of the *C. albicans* lawns, and then incubated for 48 h at 37°C.

### Determination of the Pyocyanin Levels

Strain PAO1 was grown at 37°C for 24 h in 20 ml of BSM supplemented with 40 mM succinate and mannitol, respectively. The strains PAO1 [pMMB67HE (vector control); Fürste et al., 1986] and PAO1 [pMMB*crcZ* (P*_tac_* driven constitutive expression of *crcZ*); Sonnleitner and Bläsi, 2014], respectively, were grown at 37°C for 24 h in 20 ml of BSM supplemented with 40 mM succinate. Pyocyanin was extracted from 4 ml of culture supernatant with 2 ml of chloroform, followed by an extraction with 1 ml of 0.1 M HCl. The concentration of pyocyanin was measured at A_520_, and is given as A_520_/ml of the culture supernatant.

### β-Galactosidase Assays

The β-galactosidase activities were determined as described by Miller (1972). The cells were permeabilized with 5% toluene. The β-galactosidase units in the different experiments were derived from three independent experiments.

## Results and Discussion

### CrcZ Levels During Diauxic Growth

To monitor in parallel changes in the transcriptome, translatome, and proteome during diauxic growth, strain PAO1 was grown in BSM minimal medium supplemented with 5 mM succinate (preferred carbon source) and 40 mM mannitol (less preferred carbon source). Under these conditions, the cells grew first exponentially as CCR is anticipated to prioritize metabolization of succinate (Figure 1A). After exhaustion of succinate, a lag-phase was observed after which growth resumed (Figure 1A).

In *Pae*, translational regulation by Hfq/Crc is predominantly negative during CCR (Sonnleitner and Bläsi, 2014; Kambara et al., 2018; Sonnleitner et al., 2018). After alleviation of CCR, the levels of the regulatory RNA CrcZ were expected to increase and to act as a decoy to abrogate Hfq/Crc-mediated translational repression (Sonnleitner and Bläsi, 2014; Valentini et al., 2014; Sonnleitner et al., 2018). As anticipated from studies that assessed CrcZ synthesis during growth on different carbon sources (Sonnleitner et al., 2009; Valentini et al., 2014), the levels of CrcZ RNA were the lowest during CCR, i.e., during growth on succinate (Figure 1B; T1), were augmented at the beginning of the lag-phase (Figure 1B; T2), and leveled off when growth resumed apparently on mannitol (Figure 1B; T3). As the levels of CrcZ varied during diauxic growth, the experimental set-up was deemed suitable to capture Hfq-dependent changes in the transcriptome, translatome, and proteome after relief of CCR.

### Data Analysis

Several studies on the Hfq/Crc regulon are based on RNA-seq based transcriptomics (Sonnleitner et al., 2012, 2018) or proteomics (Linares et al., 2010), which do not provide information on post-transcriptional regulation when analyzed alone. Here, we have used a multiomics approach to compare the transcriptomes, translatomes, and proteomes of cells harvested at T3 and T2 with that of T1, respectively. The same dataset was generated for the comparison of T3 with T2. The following criteria were applied for differential gene expression analysis of the multiomics data. Only annotated protein coding genes deposited in the *Pseudomonas* genome database (Winsor et al., 2016) were considered for comparison. For the Venn diagrams (Supplementary Figure S1) all genes with a low expression rate (less than 100 RNA-seq or 50 Ribo-seq reads) were disregarded. In addition, a value of *p* (adjusted for multiple testing) of 0.05 was set as a threshold for significance and the difference in FC (fold-change) had to exceed ±2 for a given gene.

We did not quantitatively compare the three datasets in terms of the correlation between gene expression- and protein fold-changes. Nevertheless, we consider this study as a useful resource for the *Pae* community interested in studying CCR regulated genes. Owing to Hfq/Crc-mediated translational repression of many genes during CCR (Corona et al., 2018; Sonnleitner et al., 2018), we primarily focused in follow-up studies on differentially regulated genes/proteins implicated in antibiotic susceptibility after relief of CCR.

### Data at a Glance

#### RNA-seq

The comparison of T2 with T1 revealed 3,302 (1,620 downregulated/1,682 upregulated) differentially abundant transcripts, whereas the comparisons of T3 with T1 and T3 with T2 revealed 1,880 (739 downregulated/1,141 upregulated) and 2,698 (782 downregulated/1,916 upregulated) differentially abundant transcripts, respectively (Supplementary Table S1; Supplementary Figure S1).

#### Ribo-seq

The comparison of T2 with T1 revealed 383 (133 downregulated/250 upregulated) differential changes in ribosomal occupancy, whereas the comparisons of T3 with T1 and T3 with T2 revealed 1,520 (715 downregulated/805 upregulated) and 1,052 (492 downregulated/560 upregulated) differential changes in ribosomal occupancy, respectively (Supplementary Table S1; Supplementary Figure S1).

#### Proteomics

When compared with the RNA-seq and Ribo-seq analyses, only ~600 proteins were found to be differentially abundant in the T2/T1, T3/T1, and T3/T2 datasets (Supplementary Table S1; Supplementary Figure S1). The low coverage of the proteome might result from the gel-based protein digestion, which is not well suited for the identification of low-abundant peptides and insoluble membrane proteins (Abhyankar et al., 2011; Mayne et al., 2016).

When compared with the RNA-seq data, the number of differentially expressed genes was lower in Ribo-seq. Discrepancies in the number of de-regulated genes in RNA-seq when compared to Ribo-seq data have been reported before (Blevins et al., 2018). Thus, our data show once more the importance of parallel application of these methods for assessment of gene expression. Notwithstanding with RNA-seq and Ribo-seq data, the number of de-regulated proteins was even lower. This observation agreed with other studies, where a poor correlation between mRNA and protein levels have been reported (Greenbaum et al., 2003; Maier et al., 2009).

As shown in the Venn diagrams (Supplementary Figure S1), with 176 genes/proteins the overlap for upregulated genes/proteins with all three approaches was most prominent when T3 was compared with T1, whereas this number decreased to 71 and 16, when T3/T2 and T2/T1, respectively, were compared. In contrast, only a few overlaps were observed for down-regulated genes/proteins in the corresponding data sets (Supplementary Figure S1).

A meta-analysis of normalized synthesis/expression of differentially abundant proteins/transcripts was therefore performed for the T3/T1 dataset. For this purpose, the genes were grouped into the corresponding KEGG pathways (Bolger et al., 2014). As shown in the heat-map, the protein, Ribo-seq and RNA-seq profiles of the T3/T1 dataset were related at the level of the KEGG pathways, although the upregulation of some degradative pathways was more apparent from the proteome data when compared to the Ribo-seq and RNA-seq analyses (Supplementary Figure S2).

### Validation of the Omics Data

Most *Pseudomonas* species lack phosphofructokinase, an enzyme which converts fructose-6-phosphate into fructose-1,6-biphosphate (Temple et al., 1998). The absence of this enzyme impedes assimilation of many carbon sources directly through the glycolytic pathway (Lessie and Phibbs, 1984). In *Pae*, the assimilation of, e.g., glucose and mannitol proceeds through the pentose phosphate (PP) and Entner-Doudoroff (ED) pathways (Dolan et al., 2020; Park et al., 2020). Hence, growth on mannitol requires a rewiring of gene expression during the lag-phase (Park et al., 2020). To validate the omics data, we first assessed the abundance of key genes/proteins required for mannitol conversion and of the PP and ED pathways. The *mtlD* and *mtlZ* genes encode enzymes required for the initial steps of mannitol catabolism (Supplementary Figure S3). The abundance of the *mtlD* and *mtlZ* transcripts increased in T2 when compared to T1. Although there was no change in the Ribo-seq reads, there was an apparent increase of the corresponding proteins. The *zwf* transcript and the encoded enzyme of the PP pathway were upregulated in T2 when compared with T1 (Supplementary Figure S3). Similarly, the key genes of the ED pathway *edaA*, *edd*, and *pgl* as well as the encoded enzymes were up-regulated in T2 when compared with T1 (Supplementary Figure S3). These results are in line with previous studies, showing that pathways required for growth on non-glycolytic carbon sources are repressed in the presence of succinate (Rojo, 2010; Dolan et al., 2020).

### Analyses of the T3/T1 Dataset Reveals an Indirect Regulation of the MexGHI-OpmD Efflux Pump by Hfq

In *Pae*, the function of CCR is not limited to the hierarchical assimilation of carbon sources. It has been reported that distinct C-sources can affect antibiotic susceptibility (Linares et al., 2010; Martinez and Rojo, 2011; Pusic et al., 2018) and other virulence traits (O'Toole et al., 2000; Pusic et al., 2016). In fact, it was shown that the absence of Hfq or titration of Hfq by the RNA CrcZ leads to an increased susceptibility towards several different classes of antibiotics (Pusic et al., 2018; Sonnleitner et al., 2020).

To search for antibiotic resistance determinants affected by CCR, we focused on genes/functions previously implicated in antibiotic susceptibility that were found to be either up- or downregulated in the RNA-seq, Ribo-seq and proteome analyses (Supplementary Figure S1). The best match between the three datasets was obtained for genes/proteins constituting the MexGHI-OpmD efflux pump, which were found to be upregulated upon growth on mannitol at T3 when compared with T1 (Table 1). The MexGHI-OpmD pump has been implicated in the export of xenobiotics, the antibiotic norfloxacin, and the dye acriflavine (Sekiya et al., 2003; Aendekerk et al., 2005). Besides, a precursor of pyocyanin, 5-methylphenazine-1-carboxylate (5-Me-PCA) was shown to be exported through MexGHI-OpmD (Sakhtah et al., 2016), which is important for self-resistance against this highly reactive compound. Like pyocyanin (Dietrich et al., 2006), 5-Me-PCA was shown to induce the *mexGHI-opmD* operon through activation of the redox-active transcription factor SoxR (Sakhtah et al., 2016).

**Table 1:** T3/T1 datasets of the transcriptome (RNA-seq), translatome (Ribo-seq), and proteome (MS) analyses of genes encoding the **(A)** MexGHI-OpmD efflux pump and **(B)** functions required for synthesis of 5-Methyl-phenazine (5-Me-PCA) and pyocyanin.

Gene name	RNA-seq		Ribo-seq		MS	
	Mannitol T3 (–CCR) vs. Succinate T1 (+CCR)					
	FC	*p* value	FC	*p* value	FC	*p* value
**A**						
*mexG*	47.36	0.00E+00	45.57	0	x	x
*mexH*	43.21	0.00E+00	21.73	1.1945E−160	3.48	8.63E−04
*mexI*	39.36	0.00E+00	14.06	2.6345E−140	4.58	3.71E−02
*opmD*	32.53	1.88E−11	32.53	1.88E−11	2.91	1.37E−02
**B**						
*phzA1*	156.03	7.20E−68	5.33	5.13E−39	x	x
*phzA2*	1023.78	8.88E−05	22.95	3.49E−96	x	x
*phzB1*	202.76	6.25E−31	11.47	7.33E−141	x	x
*phzB2*	902.22	6.21E−16	40.52	0	25.15	1.07E−02
*phzC1*	129.51	4.75E−176	8.74	4.39E−21	x	x
*phzC2*	613.74	1.54E−25	31.86	1.76E−78	x	x
*phzG1*	42.67	1.54E−23	x	x	x	x
*phzG2*	143.89	8.08E−161	x	x	x	x
*phzH*	2.49	4.00E−11	x	x	x	x
*phzM*	45.53	8.71E−04	8.79	8.81E−104	13.21	7.02E−04
*phzS*	48.73	3.36E−05	6.25	1.88E−119	6.43	9.80E−04

FC, fold-change, value of *p* ≤ 0.05. x, not efficiently translated/synthesized.

As the MexGHI-OpmD pump was shown to affect the susceptibility toward norfloxacin (Sekiya et al., 2003), we initially asked whether the observed upregulation of the *mexGHI-opmD* operon in T3 vs. T1 (Table 1) would result in a reduced susceptibility toward the antibiotic during growth in the presence of mannitol. PAO1 cells grown in BSM medium supplemented with mannitol (MIC = 1.25 μg/ml) were ~ 4-fold more resistant toward the antibiotic when compared to PAO1 grown in the presence of succinate (0.31 μg/ml) as the sole C-source.

**Figure 2:**
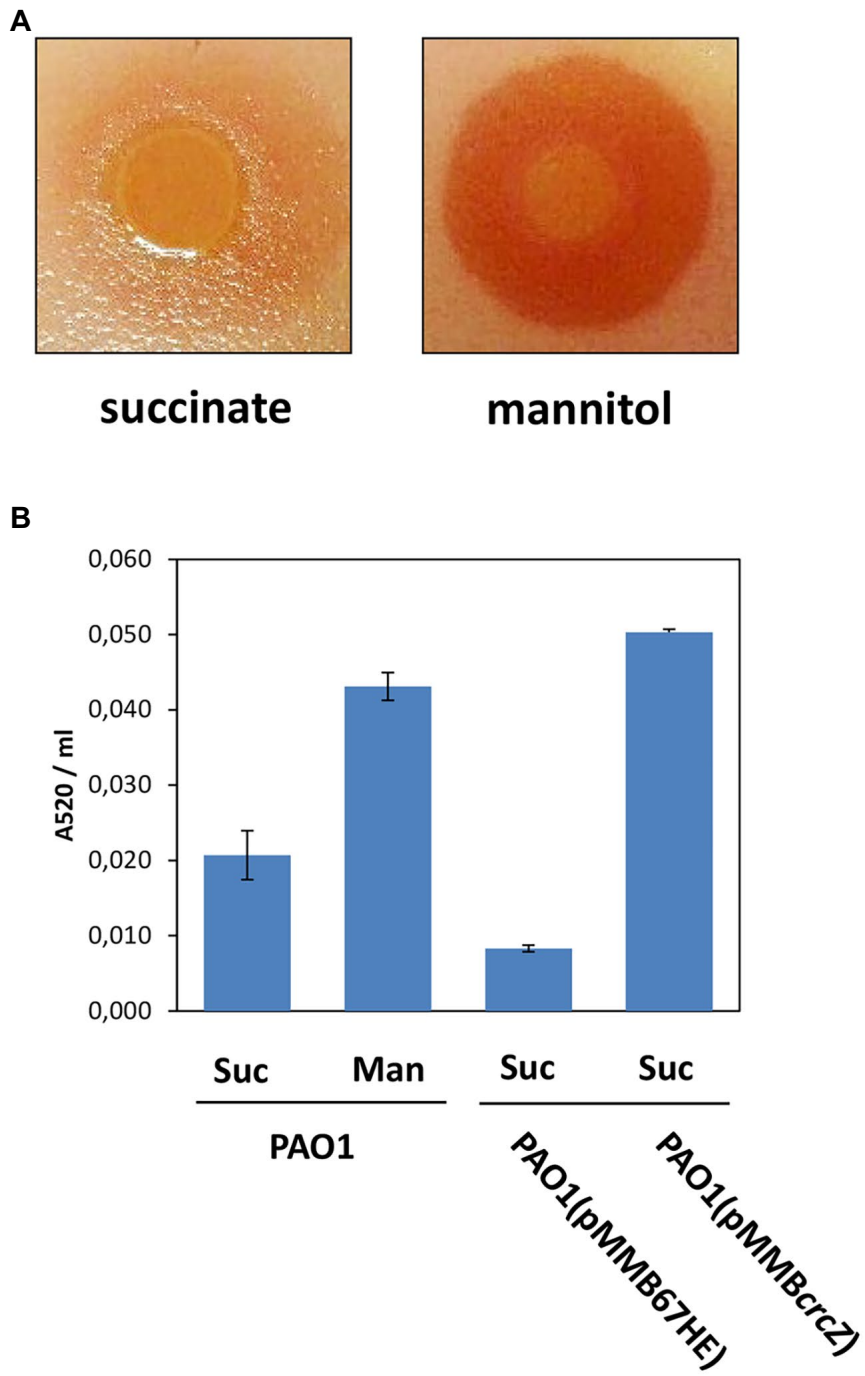
**(A)** Increased release of 5-Me-PCA results in red phenazine formation on top of *Candida albicans* lawns upon growth of PAO1 in BSM supplemented with mannitol. **(B)** Increased pyocyanin production in PAO1 and in PAO1 (pMMB*crcZ*) after growth on mannitol and upon ectopic expression of the *crcZ* gene from plasmid pMMB*crcZ* (Sonnleitner and Bläsi, 2014), respectively. The strain PAO1 (pMM67HE) harbors the parental vector of plasmid pMM*crcZ* (Sonnleitner and Bläsi, 2014). Suc, succinate; Man, mannitol. The error bars represent SDs.

As 5-Me-PCA was shown to be exported through MexGHI-OpmD in the clinical strain PA14 (Sakhtah et al., 2016), we further tested whether the increased production of the MexGHI-OpmD pump during growth on mannitol (Table 1; T3/T1) correlates with an enhanced efflux of 5-Me-PCA. For this purpose, a 5-Me-PCA bioassay was used, in which *Pae* was grown on top of *C. albicans* lawns. Under these conditions *C. albicans* can import 5-Me-PCA, which is then modified to a red fluorescent derivative (Gibson et al., 2009; Morales et al., 2010; Sakhtah et al., 2016). As shown in Figure 2A, when the cells were grown in BSM medium containing mannitol and then spotted onto the *C. albicans* lawn, a red pigmentation was visible around the colony. This was hardly visible when the cells were first grown in BSM medium supplemented with succinate. Hence, the outcome of the bioassay can be reconciled with the omics data in that the MexGHI-OpmD pump is upregulated during growth on mannitol.

In addition to the *mexGHI-opmD* operon, the genes/functions of the phenazine operons *phzA1*/2—*phzG*1/2, *phz*M and *phzS*, which are required to convert chorismate into pyocyanin (Flood et al., 1972; Mavrodi et al., 2006), were also upregulated in the T3/T1 dataset (Table 1). However, with the exception of the RNA-seq data not all genes/functions could be detected in the Ribo-seq and proteome datasets. The upregulation of the *mexGHI-opmD* and of some genes of the phenazine pathway was also observed in previous microarray and RNA-seq studies with a PAO1*∆hfq* strain (Sonnleitner et al., 2006, 2020). Moreover, an enhanced pyocyanin production was observed in a PAO1*∆hfq* strain when compared with the parental strain (Sonnleitner et al., 2003). Accordingly, when compared with growth in BSM-succinate medium, the pyocyanin production was enhanced after growth of PAO1 in BSM-mannitol medium as well as upon plasmid-mediated overproduction of CrcZ RNA (Figure 2B). Thus, the absence of Hfq as well as conditions expected to titrate Hfq resulted in higher 5-Me-PCA and pyocyanin levels.

**Figure 3:**
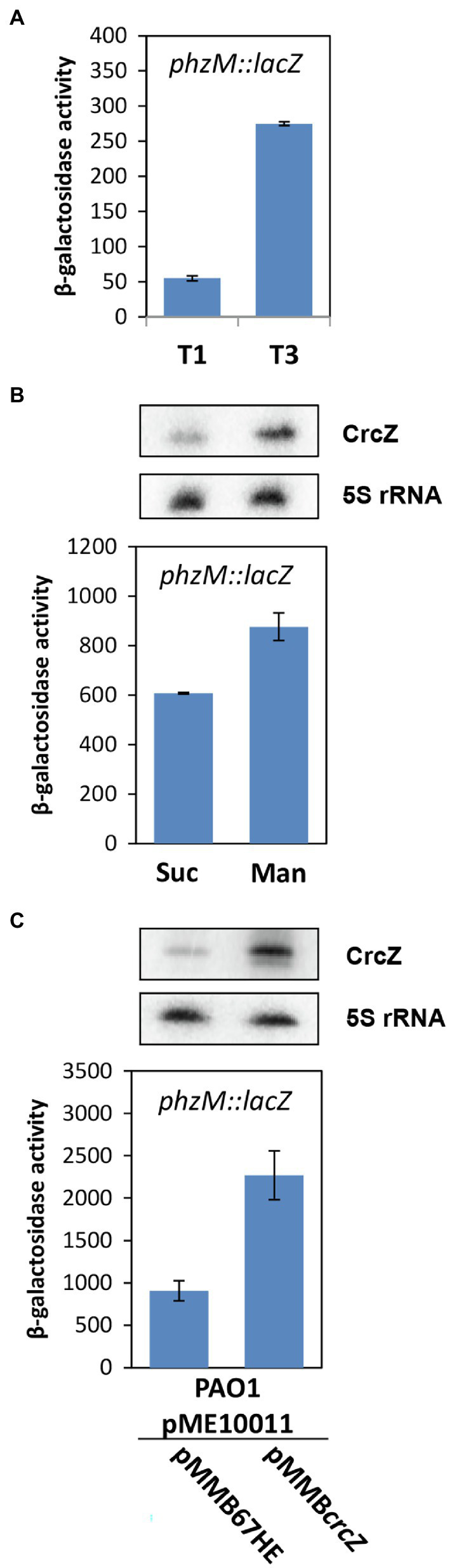
Increased levels of the RNA CrcZ result in upregulation of *phzM* translation. **(A)** Strain PAO1 (pME10011) was grown in BSM supplemented with 5 mM succinate and 40 mM mannitol. The β-galactosidase activity conferred by the translational *phzM::lacZ* reporter gene was determined at T1 and T3 (see Figure 1A), respectively. The corresponding steady state levels of the CrcZ RNA are shown in Figure 1B. **(B)** Increased *phzM::lacZ* translation during growth in BSM supplemented with mannitol. The synthesis of the PhzM-LacZ protein was assessed in strain PAO1 (pME10011) by monitoring the β-galactosidase activity after growth to an OD_600_ of 2.0 in BSM medium supplemented with succinate (Suc) and mannitol (Man), respectively. **(C)** Increased *phzM::lacZ* translation upon ectopic expression of the *crcZ* gene from plasmid pMMB*crcZ*. The synthesis of the PhzM-LacZ protein was assessed in strains PAO1 (pME10011; pMMB67HE) and PAO1 (pME10011; pMMB*crcZ*) by monitoring the β-galactosidase activity after growth to an OD_600_ of 2.0 in BSM medium supplemented with succinate. The error bars represent SDs. Top panels **(B,C)**. The steady state levels of the CrcZ RNA were determined by Northern-blot analysis when the cultures reached an OD_600_ of 2.0. 5S rRNA served as a loading control.

**Figure 4:**
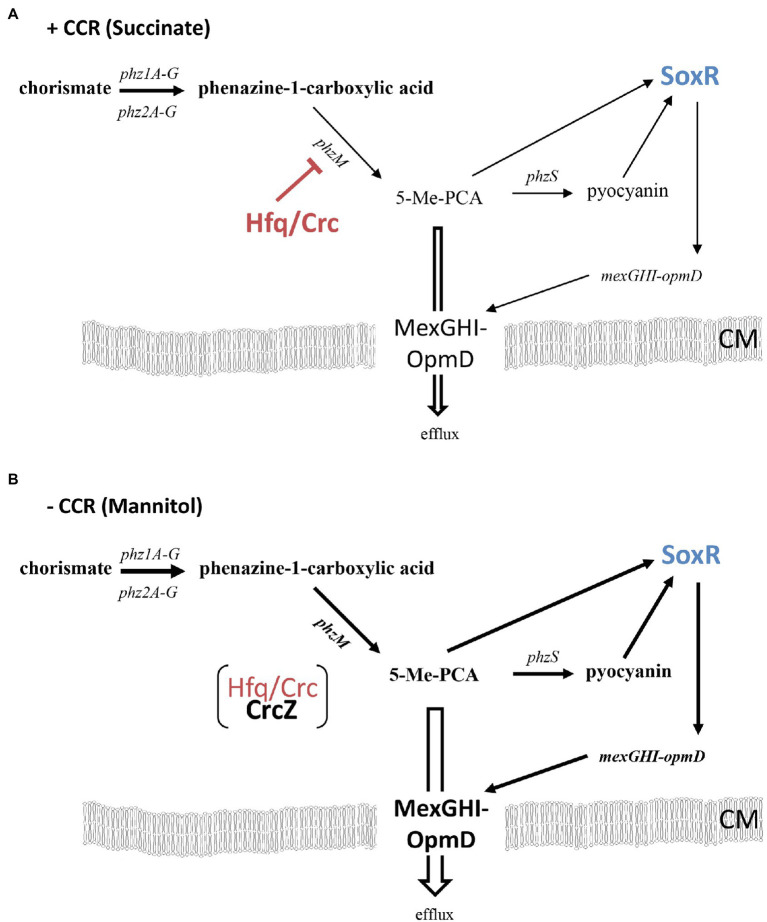
**(A)** Indirect downregulation of the *mexGHI-opmD* operon by Hfq during CCR (e.g., in the presence of succinate). The red bar indicates negative post-transcriptional regulation by Hfq/Crc of *phzM* (Sonnleitner and Bläsi, 2014), leading to moderate expression of the *mexGHI-opmD* operon (indicated by lean arrows and writing). **(B)** Increased synthesis of the *mexGHI-opmD* operon after relief of CCR (e.g., in the presence of mannitol). Titration of Hfq/Crc by CrcZ results in an increased synthesis of PhzM and hence in elevated levels of 5-Me-PCA and pyocyanin, which in turn results in activation of SoxR followed by transcriptional upregulation of the *mexGHI-opmD* operon (indicated by bold arrows and writing; Dietrich et al., 2006; Sakhtah et al., 2016). 5-Methyl-PCA efflux through MexGHI-OpmD (Sakhtah et al., 2016) protects the cells from the reactive compound. CM, cytoplasmic membrane.

How does Hfq affect 5-Me-PCA and pyocyanin synthesis? The *phzM* gene, encoding the *N*-methyltransferase presumed to convert PCA to 5-Me-PCA (Parsons et al., 2007), was shown to be negatively regulated by Hfq at the post-transcriptional level in *Pae* M18 (Wang et al., 2012). In addition, owing to Hfq-binding motifs overlapping with the ribosome binding site, the *phzM* gene was shown to be translationally repressed by Hfq/Crc in strain PAO1 (Sonnleitner and Bläsi, 2014). Moreover, the *phzM* mRNA was more abundant after the anticipated titration of Hfq by CrcZ at T2 and T3, when compared with T1 (Supplementary Table S1; Table 1). Thus, we hypothesized that translational repression of *phzM* by Hfq/Crc in the presence of succinate leads to low levels of 5-Me-PCA as observed in the bioassay (Figure 2A). On the other hand, de-repression of *phzM* during growth on mannitol, i.e., after synthesis of CrcZ and relief of CCR, could explain the elevated levels of 5-Me-PCA (Figure 2A). To substantiate this hypothesis, we made use of a translational *phzM::lacZ* reporter gene mounted on plasmid pME10011 (Huang et al., 2012) and determined the synthesis of the PhzM-LacZ protein by measuring the β-galactosidase activity of strain PAO1 (pME10011) at T1 and T3 during diauxic growth (see Figure 1). As shown in Figure 3A, the PhzM-LacZ production was ~5-fold increased at T3 when compared with T1. In addition, the synthesis of the PhzM-LacZ protein was assessed in strain PAO1 (pME10011) after growth to an OD_600_ of 2.0 in BSM medium supplemented with succinate and mannitol, respectively. When compared with growth in the presence of succinate, *phzM::lacZ* translation and CrcZ levels were increased in the presence of mannitol (Figure 3B). Furthermore, the strains PAO1 (pMMB*crcZ*; pME10011) and PAO1 (pMMB67HE; pME10011) were grown to an OD_600_ of 2.0 in BSM medium supplemented with succinate. When compared with strain PAO1 (pMMB67HE; pME10011), harboring the parental vector of plasmid pMMB*crcZ* (Sonnleitner and Bläsi, 2014), ectopic expression of *crcZ* in strain PAO1 (pMMB*crcZ*; pME10011) resulted in increased PhzM-LacZ production (Figure 3C). Hence, in contrast to Hfq/Crc-mediated translational repression of *phzM* during growth on succinate (Figure 4A), CrcZ-mediated titration of Hfq during growth on mannitol and the resulting upregulation of the *phzM* gene can explain the observed increase in 5-Me-PCA and pyocyanin levels (Figure 2). On the other hand, the 5-Me-PCA-dependent activation of SoxR (Sakhtah et al., 2016) can in turn explain the observed upregulation of the *mexGHI-opmD* operon after alleviation of CCR (Figure 4B). It is also worth mentioning that two other SoxR regulated transcripts, PA*2274* and PA*3718* (Palma et al., 2005), show a higher abundance after alleviation of CCR (Supplementary Table S1).

## Conclusion

In our experimental setup, we cannot exclude other factors such as an increase of byproducts of cellular metabolism or quorum sensing that might impact phenanzine synthesis (Lee and Zhang, 2015; Higgins et al., 2018), and thus *mexGHI-opmD* expression (Sakhtah et al., 2016) during diauxic growth. However, as *phzM* translation was de-repressed in a PAO1*∆hfq* deletion strain and CrcZ was shown to titrate Hfq (Sonnleitner and Bläsi, 2014), and induction of the *phzM::lacZ* reporter gene correlated with increased CrcZ levels (Figures 3B,C), a simple rationale for the observed upregulation of the *mexGHI-opmD* operon after relief of CCR is the sequestration of Hfq by the RNA CrcZ, leading to elevated synthesis of the PhzM enzyme.

We have previously shown that CrcZ-mediated titration of Hfq generally enhances the sensitivity toward antibiotics (Pusic et al., 2018). For instance, CrcZ-mediated abrogation of negative translational regulation by Hfq of the *oprD* and *opdP* porin genes results in increased carbapenem susceptibility (Sonnleitner et al., 2020). Although controlled synthesis of CrcZ can provide a means to (re)sensitize *P. aeruginosa* to certain antibiotics it apparently can also decrease the susceptibility to antibiotics, when relief of Hfq-mediated negative regulation results in the synthesis of metabolites, which in turn stimulate synthesis of resistance determinants, such as the *mexGHI-opmD* operon. Hence, distinct Hfq-dependent regulatory circuits either leading to an increased or a decreased susceptibility towards individual antibiotics should be considered when using sugars and other metabolites as adjuvants in antibiotic treatment of *Pae* infections (Meylan et al., 2017).

## Data Availability Statement

The raw RNA-seq and Ribo-seq data were deposited in the ENA under accession number PRJEB52258.

## Author Contributions

UB, MR, ES, and WW: conceived and designed the experiments. MR, EN, and ES: performed the experiments. FA, UB, MR, ES, MW, and WW: analyzed the data. All authors contributed to the article and approved the submitted version.

## Funding

The work was supported by the Austrian Science Fund (www.fwf.ac.at/en) through project P28711-B22 (UB and ES). MR was supported through the FWF funded doctoral program RNA-Biology W-1207.

## Conflict of Interest

The authors declare that the research was conducted in the absence of any commercial or financial relationships that could be construed as a potential conflict of interest.

## Publisher’s Note

All claims expressed in this article are solely those of the authors and do not necessarily represent those of their affiliated organizations, or those of the publisher, the editors and the reviewers. Any product that may be evaluated in this article, or claim that may be made by its manufacturer, is not guaranteed or endorsed by the publisher.

## Footnotes

^1^ http://www.csf.ac.at

^2^ https://www.uniprot.org/taxonomy/208964
