# Supplementary material for: Rewiring of Gene Expression in Pseudomonas aeruginosa During Diauxic Growth Reveals an Indirect Regulation of the MexGHI-OpmD Efflux Pump by Hfq
Source: Front Microbiol. 2022 Jun 23;13:919539. doi: 10.3389/fmicb.2022.919539 (PMC9272787; doi:10.3389/fmicb.2022.919539)
Supplement: Supplementary file 2 [file Data_Sheet_2.pdf]

Supplementary Material

Rewiring of Gene Expression in *Pseudomonas aeruginosa* During Diauxic Growth Reveals an Indirect Regulation of the MexGHI-OpmD Efflux Pump by Hfq

Marlena Rozner, Ella Nukarinen, Michael T. Wolfinger, Fabian Amman, Wolfram Weckwerth, Udo Bläsi* and Elisabeth Sonnleitner*

*Correspondence:

udo.blaesi@univie.ac.at

elisabeth.sonnleitner@univie.ac.at

1 Supplementary Figures and Tables

1.1 Supplementary Tables

Supplementary Table 1 Compilation of the T3/T1, T2/T1 and T3/T2 RNA-seq, Ribo-seq and translatome datasets. The numbers of transcripts, ribosome footprints and peptides with a fold-change ≥ 2 or ≤ 2 and a multiple testing adjusted *p*-value ≤ 0.05 are listed in bold, whereas values below these thresholds are shown in pale writing.

1.2 Supplementary Figures

A

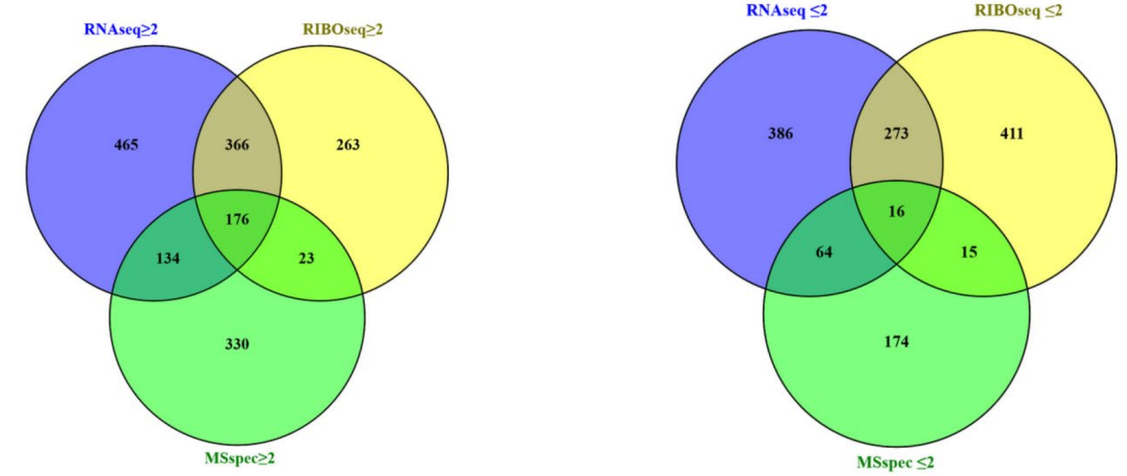
RNAseq≥2
RIBOseq≥2
465
366
263
176
134
23
330
MSspec≥2
RNAseq ≤2
RIBOseq ≤2
386
273
411
16
64
15
174
MSspec ≤2

B

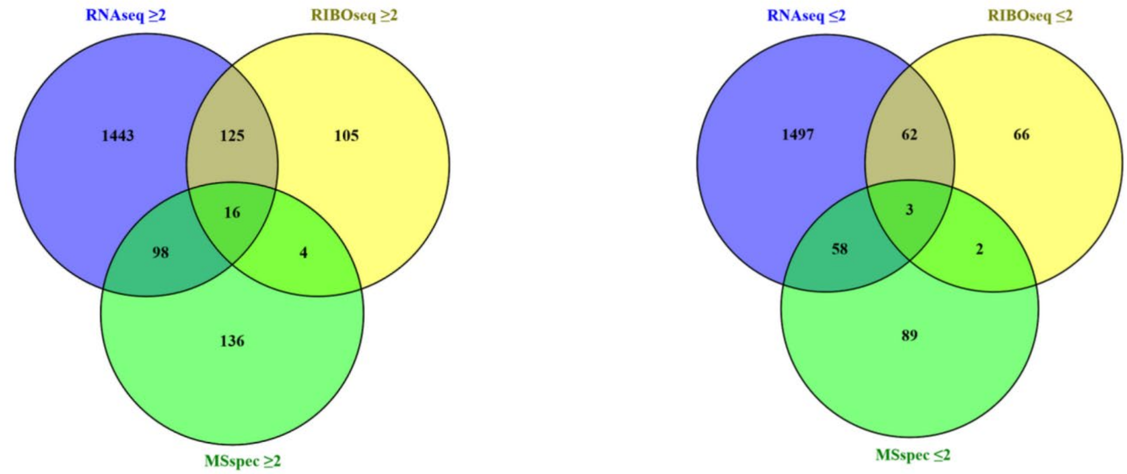
RNAseq ≥2
RIBOseq ≥2
1443
125
105
16
98
4
136
MSspec ≥2
RNAseq ≤2
RIBOseq ≤2
1497
62
66
3
58
2
89
MSspec ≤2

C

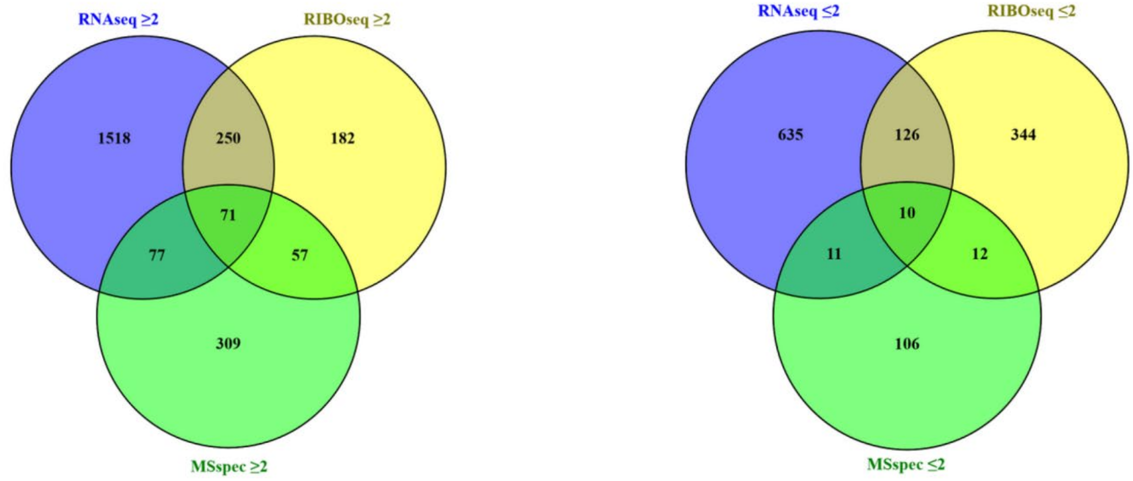
RNAseq ≥2
RIBOseq ≥2
1518
250
182
71
77
57
309
MSspec ≥2
RNAseq ≤2
RIBOseq ≤2
635
126
344
10
11
12
106
MSspec ≤2

Supplementary Figure S1 Venn diagrams showing the number of transcripts with increased (left panels) or decreased (right panels) abundance in RNA-seq, Ribo-seq and the MS data obtained after analysis of the following datasets: **(A)** T3 *vs* T1 (mannitol uptake vs succinate uptake); (**B**) T2 *vs* T1; (**C**) T3 *vs* T2; For significance only a FC ≥ 2 or ≤ 2 and a multiple testing adjusted *p*-value ≤ 0.05 are considered for the RNA-seq, Ribo-seq and MS data. The corresponding transcripts, ribosomal footprints and MS data with increased or decreased abundance are listed in **Supplementary Table 1**.

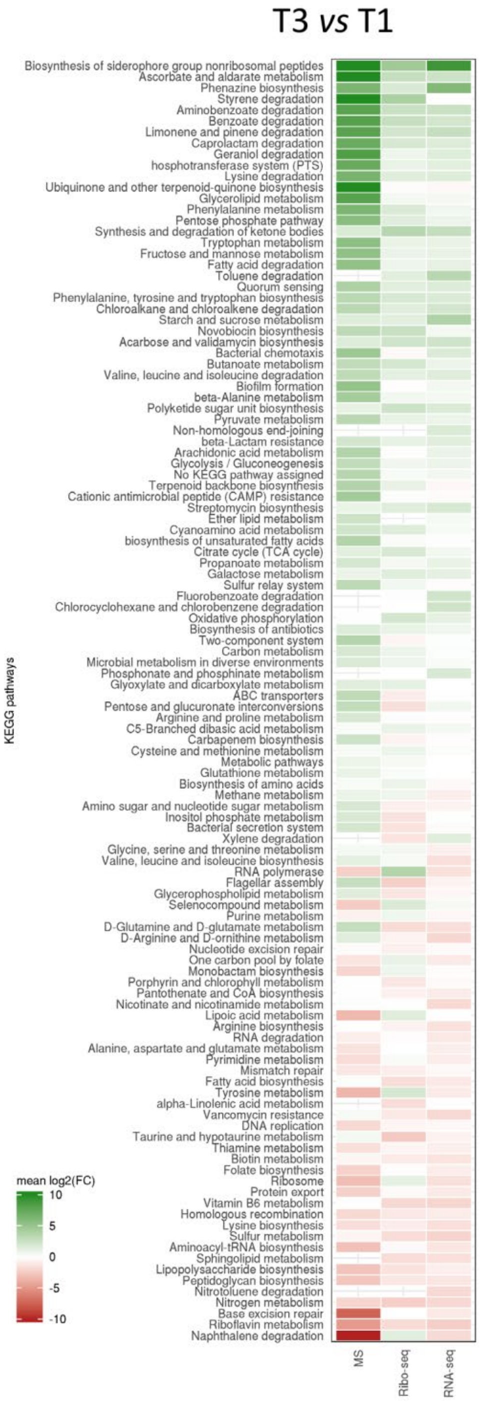

Supplementary Figure S2 Meta-analysis of the dataset T3 *vs* T1 of normalized synthesis / expression of differentially abundant proteins resulting from the MS data and transcripts revealed by Ribo-seq and RNA-seq, respectively. The genes are grouped into the corresponding pathways (http://www.kegg.jp/kegg-bin/show_organism?org=pae). For each group the averaged $\log_2$ fold changes of significantly modulated members are shown. The colour code shown in the scale at the left denotes $\log_2$-fold changes. Red indicates an overall decrease and green indicates an overall increase in the protein and mRNA levels in a particular pathway.

A

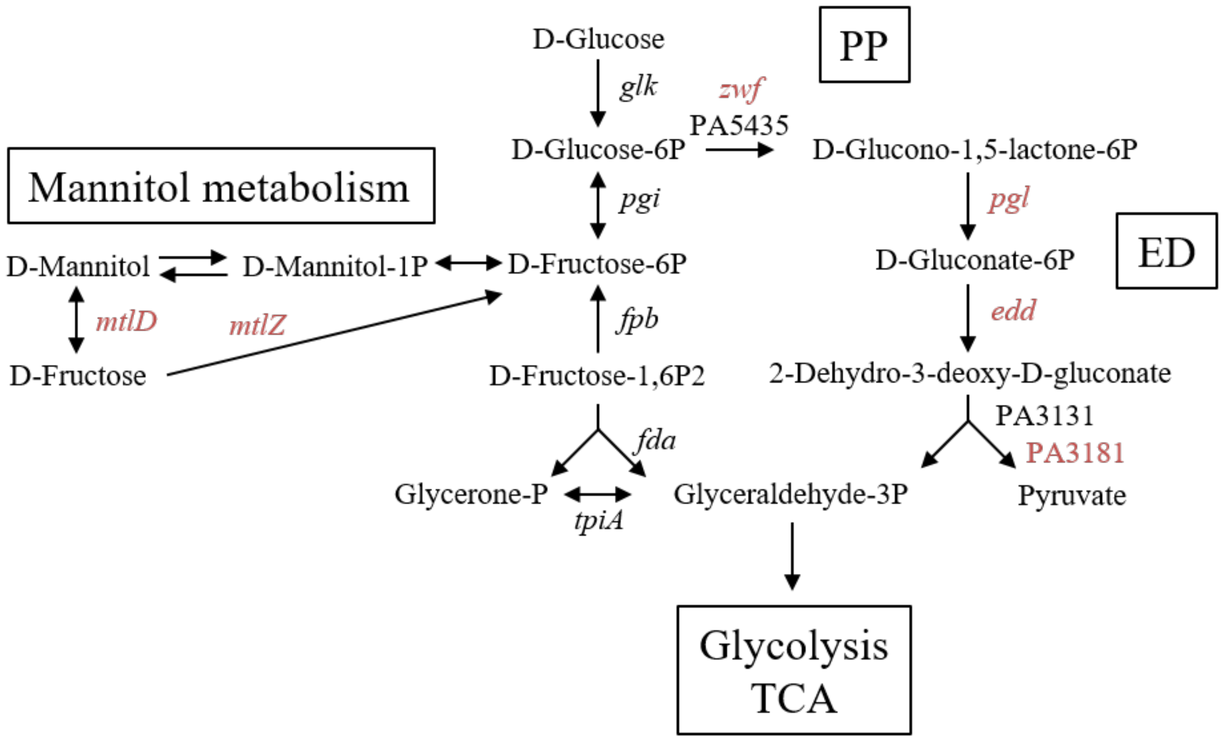

B

		T2 vs T1					
PA number	gene name	RNA-seq		Ribo-seq		MS	
		FC	p-value	FC	p-value	FC	p-value
**Pentose phosphate pathway**							
PA3183	*zwf*	25.4	0.00E+00	1.91	2.94E-17	4.99	3.38E-04
**Entner–Doudoroff pathway**							
PA3181	*edaA*	82.5	0.00E+00	5.84	6.37E-54		
PA3194	*edd*	14.0	0.00E+00	2.28	4.86E-31	-1.22	4.56E-01
PA3182	*pgl*	85.6	0.00E+00	3.21	3.07E-31	7.09	3.70E-04
**Mannitol metabolism**							
PA2344	*mtlD*	17.2	0.00E+00	1.02	7.69E-01	1.42	5.73E-01
PA2344	*mtlZ*	8.14	0.00E+00	1.03	7.06E-01	1.61	5.95E-01

Supplementary Figure S3 (**A**) *Pae* utilizes the pentose phosphate (PP) and the Entner-Doudoroff (ED) pathways for metabolization of different carbohydrates, such as mannitol (Dolan et al., 2020; Park et al., 2020). Key enzymes for these pathways are highlighted in red. (**B**) RNA-seq, Ribo-seq and MS data for the functions highlighted in red in (**A**).
